# Evaluation of Norovirus Reduction in Environmentally Contaminated Pacific Oysters During Laboratory Controlled and Commercial Depuration

**DOI:** 10.1007/s12560-021-09464-2

**Published:** 2021-03-02

**Authors:** Agnieszka Rupnik, William Doré, Leon Devilly, James Fahy, Amy Fitzpatrick, Wiebke Schmidt, Kevin Hunt, Francis Butler, Sinéad Keaveney

**Affiliations:** 1 Marine Institute, Rinville, Oranmore, Ireland; 2 Centre for Food Safety, University College Dublin, Dublin, Ireland

**Keywords:** Depuration, Human norovirus, Risk management, Oysters, RT-qPCR, ISO 15216-1

## Abstract

Norovirus contamination of oysters is the lead cause of non-bacterial gastroenteritis and a significant food safety concern for the oyster industry. Here, norovirus reduction from Pacific oysters (*Crassostrea gigas),* contaminated in the marine environment, was studied in laboratory depuration trials and in two commercial settings. Norovirus concentrations were measured in oyster digestive tissue before, during and post-depuration using the ISO 15216-1 quantitative real-time RT-PCR method. Results of the laboratory-based studies demonstrate that statistically significant reductions of up to 74% of the initial norovirus GII concentration was achieved after 3 days at 17–21 °C and after 4 days at 11–15 °C, compared to 44% reduction at 7–9 °C. In many trials norovirus GII concentrations were reduced to levels below 100 genome copies per gram (gcg^−1^; limit of quantitation; LOQ). Virus reduction was also assessed in commercial depuration systems, routinely used by two Irish oyster producers. Up to 68% reduction was recorded for norovirus GI and up to 90% for norovirus GII reducing the geometric mean virus concentration close to or below the LOQ. In both commercial settings there was a significant difference between the levels of reduction of norovirus GI compared to GII (*p* < 0.05). Additionally, the ability to reduce the norovirus concentration in oysters to < LOQ differed when contaminated with concentrations below and above 1000 gcg^−1^. These results indicate that depuration, carried out at elevated (> 11 °C) water temperatures for at least 3 days, can reduce the concentration of norovirus in oysters and therefore consumer exposure providing a practical risk management tool for the shellfish industry.

## Introduction

Norovirus infections are the most common cause of non-bacterial gastroenteritis worldwide (Marshall et al. 2003; Marshall and Bruggink 2011). Filter-feeding bivalve molluscan shellfish such as mussels, clams and oysters can become contaminated with human norovirus when grown in areas impacted by human sewage discharges. Such shellfish present a recognised public health risk when consumed raw or lightly cooked (Bellou et al. 2013) and regulations exist throughout the world to manage their production. In Europe, regulatory controls predominantly centre around the sanitary classification of harvesting areas into three categories, A, B or C, based on increasing *Escherichia coli* concentrations (Anonymous 2004, 2019). Each classification category calls for differing degrees of post-harvest treatment: from no additional treatment for shellfish harvested from class A waters, to relay and depuration for shellfish grown in class C areas. One of the most widely practiced post-harvest treatments is depuration, whereby bivalve shellfish undergo self-purification in land-based tanks of clean seawater. The process was originally designed in the beginning of the twentieth century to prevent bacterial illness associated with shellfish consumption.

In order to comply with EU regulations shellfish harvested from class B areas, accounting for approximately 60% of overall oyster production across the EU (EFSA 2019) must be depurated prior to going to market. Despite these regulatory obligations virtually eliminating bacterial illness associated with bivalve shellfish, there are many reports of such product causing outbreaks of viral illness (Morse et al. 1986; Chalmers and McMillan 1995; Lee et al. 1999; Kingsley et al. 2002; Baker et al. 2010; LeBlanc et al. 2016). In particular, such outbreaks have been associated with the consumption of oysters (Le Guyader et al. 2010; Westrell et al. 2010; Alfano-Sobsey et al. 2011; Rajko-Nenow et al. 2013). Oysters harvested from A classified areas, where post-harvest treatment is not mandatory, have also been associated with outbreaks of illness (Doré et al. 2010). Oysters present additional risks due to a number of factors such as being grown in intertidal areas often impacted by sewage and being consumed raw or only lightly cooked. This has resulted in an increasing number of commercial oyster producers in Ireland and elsewhere that harvest from A classified production areas to include depuration as an extra step in their HACCP (Hazard Analysis and Critical Control Point) or risk management procedures (Rupnik et al. 2018).

Minimum time and water temperature used in commercial depuration are not stipulated in EU regulation. In Ireland, it is recommended that depuration should be carried out for a minimum of 42 h with a water temperature of no less than 8 °C (Anonymous 2018). This treatment has been shown to consistently reduce *E. coli* concentrations to below the regulatory limit of 230 MPN/100 g, but not to reduce norovirus to the same extent (Schwab et al. 1998; McLeod et al. 2009). However, some studies have indicated that depuration time and seawater temperature are both factors that may influence virus reduction during bivalve shellfish depuration (Lees et al. 2010). The potential for norovirus reduction in oysters during enhanced depuration procedures was identified previously by this group (Rupnik et al. 2018) prompting this study to investigate norovirus reduction in oysters under controlled laboratory conditions.

Artificial contamination of shellfish with viral pathogens is a common approach to studying depuration processes and has been described previously (Muniain-Mujika et al. 2002; Choi and Jiang 2005; Nappier et al. 2008; McLeod et al. 2009; Polo et al. 2014b). As shellfish are known to efficiently bioaccumulate viruses in as little as several hours (Flannery et al. 2012; Souza et al. 2013, 2018; Pilotto et al. 2019), this approach allows for a rapid generation of animals contaminated with one or multiple viral strains. Concentrations as high as 10^11^ genome copies per gram (gcg^−1^) of murine norovirus (MNV1) were achieved during 24h bioaccumulation in *C. gigas* by Pilotto et al. (2019), whereas both GI and GII human norovirus genogroups, simultaneously bioaccumulated in *C. gigas* oysters to 10^6^ gcg^−1^ (Maalouf et al. 2011). Maximum reductions of the norovirus concentrations in such artificially contaminated shellfish were previously reported in the region of 1–1.2 log_10_ unit (equivalent to 90–94% reduction) and based on that evidence the process was deemed ineffective (McLeod et al. 2017). However, the initial viral loads in such conditioned animals would be considered to be much higher than those seen in oysters from classified production areas across Europe as confirmed by the EFSA baseline survey conducted between November 2016 and October 2018 (EFSA 2019). Much less is therefore known about the efficacy of norovirus reduction of environmentally contaminated oysters from classified production areas under controlled laboratory conditions or in commercial operations. Evidence for viral reduction during modified depuration procedures was reported by (Doré et al. 1998) using FRNA bacteriophage where a significant reduction in FRNA bacteriophage was observed when water temperature was increased to 18 °C. The introduction of quantitative real-time PCR for the detection and quantification of norovirus in bivalve shellfish, particularly the method described in ISO 15216-1:2017 (Anonymous 2017), has been an important step in the area of risk management of norovirus. This tool has allowed better understanding of the relative differences in norovirus concentrations when applying risk management procedures (Rupnik et al. 2018), as well as understanding the concentrations of norovirus found in classified production areas across the EU (EFSA 2019).

As far as the authors are aware, this is the first study using the ISO 15216-1 standard method based on real-time RT-PCR to measure norovirus RNA concentrations in oysters contaminated in their natural environment during controlled laboratory depuration studies and enhanced commercial depuration procedures. Our aim was to provide evidence to support the hypothesis that appropriate depuration conditions can be successfully used in the commercial setting as part of risk management measures to reduce the risk to consumer by decreasing the norovirus concentrations in market-ready shellfish.

## Materials and Methods

### Oysters

Triploid Pacific oysters (*Crassostrea gigas*) contaminated with norovirus in their growing areas were used in all laboratory depuration trials. Oysters from a commercial classified production area were monitored for norovirus GI and GII contamination on a weekly basis and results of the monitoring were used to schedule the depuration experiments. Once the norovirus concentrations increased to, or above, 300 gcg^−1^ a batch of oysters were harvested, washed and transported to the laboratory within 27 h under temperature-controlled conditions (< 15 °C). On receipt in the laboratory, oysters were stored in dry, cool conditions and used in depuration trials within 24 h.

### Laboratory Depuration Trials

Two 0.6m^3^ tanks (Depur Systems, UK) were filled with 400–450 L potable mains water and 15 kg of Seamix Artificial Sea Water salt mix (NaCl 66.1%; MgSO_4_ 16.3%; MgCl_2_ 12.7%; CaCl_2_ 3.3% and KCl 1.6%; Peacock Salt, UK) was added to each and stirred until fully dissolved. The amount of salt added increased water salinity to between 28 and 32ppt mimicking that of the estuary. This artificial seawater was circulated in the tank overnight to fully equilibrate before depuration experiments commenced. Water temperature was maintained within 1 °C of target temperature with a combination of 300 W aquarium heaters placed directly in the tank and an externally located 750 W water chiller (D&D, DC750). Bar sprinklers located above one side wall of the tank were used for aeration and water flow was maintained using an external pump at 2000–2200 L/h. One 36 W UV-C lamp fitted inline on each tank was used to sterilise the circulating water. Dissolved oxygen levels were measured once daily using a hand-held OxyGuard device. Oysters were placed loosely in shallow plastic trays, approximately 70–80 animals per tray. In total, four trays per tank (between 280 and 320 oysters) were placed in each depuration tank and subjected to depuration. Triplicate samples of 12 oysters were collected at commencement of the experiment (Day 0) and at 24-h intervals thereafter (Days 1–7). Oysters were stored at 4 °C for up to 24 h before further processing. A full depuration cycle was run for 7 days (approx.168 h) after which the tanks were drained, cleaned, and filled with freshly prepared artificial sea water before the next experiment.

### Shelf Life Test

Forty-five oysters remaining in the depuration tanks after completion of the laboratory trials at 8 °C and 18 °C were placed on shallow trays and refrigerated (5 °C) for 4 weeks. Once a week, oysters were inspected and any gaping or unresponsive to percussion animals were counted and disposed of.

### Commercial Depuration

Paired samples, pre- and post-depuration, were collected from two separate oyster producers between January 2018 and December 2019 (Producer A) and February 2018 to April 2019 (Producer B). Producer A operates in a class A area, whilst Producer B operates in an area with a seasonal A classification during December through to March and class B for the rest of the year. Depuration is routinely used in both facilities with Producer A typically depurating oysters for 7 days with an average water temperature of 14 °C (ranging from 12 to 16 °C between the depuration cycles). Producer B performs depurations typically for 72 h at an accurately controlled stable 18 °C. Twenty-four and 22 depuration cycles carried out by Producer A and B, respectively, were examined. Pre-depuration oyster samples were collected on the day depuration commenced and were followed by a second, paired sample taken after the completion of the depuration process. Each sample consisted of 12 live animals that were transported into the laboratory under chilled conditions (< 15 °C) within 27 h (Producer A) or 5 h (Producer B). Upon arrival, oysters were stored at 4 °C for up to 24 h before processing. When evaluating the commercial depuration processes, both norovirus genogroups were quantified in the pre-depuration samples. Samples in which the virus concentration was greater than 150 gcg^−1^ for at least one genotype were selected and subsequently followed with a sample taken after completion of depuration. This allowed for concurrent analysis of the effect of depuration on both norovirus genotypes in oysters.

### Norovirus Detection in Oysters

#### Preparation of Oyster Samples for Norovirus Analysis

Oysters were analysed for the presence of norovirus GI and GII in accordance with ISO 15216-1:2017 (Anonymous 2017). Briefly, oysters were cleaned under running potable water. Ten oysters per sample were shucked and the digestive tissues (DT) dissected out. The dissected DT was finely chopped using a sterile razor blade and mixed well. Two grams of DT were then spiked with 10 µl of the internal process control (IPC) virus (Mengo virus strain MC_0_) for evaluation of virus extraction efficiency similar to that described by (Costafreda et al. 2006) and treated with 2 ml Proteinase K (100 µg ml^−1^). Samples were incubated at 37 °C for 60 min with shaking at 150 rpm followed by 15 min at 60 °C. Finally, after centrifugation at 3000×*g* for 5 min, supernatants were retained for RNA extraction.

#### Viral RNA Extraction

RNA was extracted from 500 µl of the DT supernatants using NucliSENS® magnetic extraction reagents (bioMérieux) and the NucliSENS® MiniMAG® extraction platform and eluted into 100 µl of elution buffer. RNA extracts were stored at − 80 °C until the RT-qPCR analysis was conducted. RNA was also extracted from 10 µl of the IPC sample for evaluation of extraction efficiency. A single negative extraction control (water only) was processed alongside the oyster samples.

#### Determination of the Norovirus Concentration Using One-Step RT-qPCR

Oysters were analysed for the norovirus concentrations using standardised quantitative real-time reverse transcription PCR (RT-qPCR) (Anonymous 2017). RT-qPCR analysis was carried out using the Applied Biosystems AB7500 instrument (Applied Biosystems, Foster City, CA) and the RNA Ultrasense one-step RT-qPCR system (Invitrogen). The reaction was prepared by combining 5 µl of the extracted RNA sample and 20 µl of the reaction mix containing 500 nM forward primer, 900 nM reverse primer, 250 nM sequence specific probe,1 × ROX reference dye and 1.25 µl of enzyme mix. Previously described primers QNIF4 (da Silva et al. 2007), NV1LCR (Svraka et al. 2007) and TM9 probe (Hoehne and Schreier 2006) were used for the detection of norovirus GI, and QNIF2 (Loisy et al. 2005), COG2R (Kageyama et al. 2003) and QNIFS probe (Loisy et al. 2005) were used for the detection of norovirus GII. The Mengo110, Mengo209 primers and Mengo147 probe were used in IPC assay (Pintó et al. 2009). The 96-well optical reaction plate was incubated at 55 °C for 60 min, 95 °C for 5 min, and then 45 cycles of PCR were performed, with 1 cycle consisting of 95 °C for 15 s, 60 °C for 1 min, and 65 °C for 1 min. All samples were analysed for norovirus GI and GII in duplicate. All control materials used in the RT-qPCR assays were prepared as described by Flannery et al. (2012). To enable quantification of norovirus RNA in copies per µl, a log_10_ dilution series of the norovirus GI and GII DNA plasmids (ranging from 1 × 10^1^ to 1 × 10^5^ copies/µl) were included in duplicate on each RT-qPCR run. The number of RNA copies in norovirus-positive samples was determined by comparing the *C*_*T*_ value to the standard curves. The final concentration was then adjusted to reflect the volume of sample analysed and expressed as the number of detectable virus genome copies per gram of DT. The presence of inhibitors was checked by spiking an additional 5 µl of each sample RNA with 1 µl of either norovirus GI or norovirus GII external control RNA (ECRNA; 10^5^ RNA transcripts/µl). The threshold cycle (*C*_*T*_) value obtained for samples spiked with the ECRNA was compared to the results obtained in the absence of the sample (5 µl of water used instead) and used to estimate RT-PCR inhibition expressed as a percentage. In accordance with ISO 15216-1:2017. Oyster samples with RT-PCR inhibition below the 75% were accepted for inclusion in this study. Extraction efficiency was assessed by comparing the *C*_*T*_ value of the sample spiked with IPC virus to a standard curve obtained by preparing log_10_ dilutions of the RNA extracted from 10 µl Mengo virus and was subsequently expressed as percentage extraction efficiency. Samples with the extraction efficiency greater than 1% were accepted for inclusion in this study (Anonymous 2017). No template controls (water only) and negative extraction controls (blank sample carried through the RNA extraction step) were included in each RT-PCR analysis to control for cross-contamination.

### Analysis of Results

#### LOD and LOQ

The limits of detection (LOD) and quantification (LOQ) for Norovirus GI and GII were established based on European Union Reference Laboratory (EURL) for monitoring the bacteriological and viral contamination of bivalve molluscs guidance using dilution series of oyster digestive gland material contaminated with both, norovirus GI and GII (CEFAS 2016). The LOD and LOQ for norovirus GI and GII was determined as 20 and 100 gcg^−1^, respectively. For statistical analysis, and in order to facilitate geometric mean calculation, samples in which norovirus was not detected were assigned a value of 10 gcg^−1^ (half of the LOD) and those that tested positive below 100 gcg^−1^ (< LOQ) were assigned a value of 50 gcg^−1^ (half of LOQ).

#### Relative Concentrations

Due to variances in starting norovirus concentration, and to allow for comparison between experiments and statistical analysis, all concentrations were converted to values relative to Day 0 (initial load), as follows: *C*_Ri_ = *C*_i_/*C*_0_, where *C*_Ri_ – Relative concentration of sample *i*; *C*_i_ –norovirus concentration gcg^−1^ obtained for sample *i* using RT-qPCR and *C*_0_ –geometric mean of initial load concentration in a given experiment. For example, 2023, 1781 and 1459 norovirus GII gcg^−1^ were detected in samples A, B and C collected at Day 0 giving a geometric mean of 1739 norovirus GII gcg^−1^. Therefore, the relative result for sample A = 2023 gcg^−1^ /1739 gcg^−1^ = 1.163; sample B = 1781 gcg^−1^ /1739 gcg^−1^ = 1.024 and sample C = 1459 gcg^−1^ /1739 gcg^−1^ = 0.839. This principle was applied to all samples.

#### Assigning of Data for Laboratory Depuration Trials

Norovirus test results were assigned into 3 separate groups based on the water temperature used in a given depuration trial: Low—depuration experiments carried out at 8 ± 1 °C (*n* = 5); Medium—depuration experiments carried out at 12 or 14 °C (± 1 °C; *n* = 3) and High—depuration experiments carried out at 18 or 20 °C (± 1 °C; *n* = 7).

#### Statistical Analysis

R (version 3.6.0) using the libraries ‘car’ and ‘PMCMR’ was used for statistical analysis. The normality of the data was tested by performing the Shapiro–Wilk test and the Levene test was used to evaluate the homogeneity of variances. To evaluate the differences between three temperatures conditions (low, medium and high) during the depuration study a Kruskal–Wallis rank ANOVA with the pairwise test for multiple comparison of mean rank sums (Dunn’s test) as posthoc tests was used. To test the difference of norovirus concentrations during commercial depuration the Mann–Whitney test was applied. The significance level for all statistical analysis was set at *p* ≤ 0.05.

## Results

### Laboratory Depuration Trials

Stable conditions in the depuration tanks were maintained throughout each experiment with dissolved oxygen (DO_2_) levels in excess of 80% in all trials. Norovirus GI concentrations detected in *C. gigas* before depuration were either below or close to the limit of quantification (LOQ) of the test (100 gcg^−1^) and therefore were excluded from further analysis in this study. Before depuration, norovirus GII concentrations in the environmentally contaminated oysters ranged between 178 and 16,426 norovirus GII gcg^−1^ with a geometric mean of 906 norovirus GII gcg^−1^ (Table 1).

**Table 1:** Norovirus GII concentrations measured in Pacific oysters during laboratory depuration trials

Experiment	Water temperature [°C]	Time point [days]/norovirus GII concentration [gcg^−1^]							
		0	1	2	3	4	5	6	7
1	8	16,426	15,792	7803	7463	10,742	5443	11,210	7558
2	8	290	310	284	142	< LOQ	249	147	225
3	8	5573	14,505	7428	5981	2965	3334	3548	2293
4	8	1739	1970	2309	1601	2122	4798	2087	3020
5	8	850	1247	956	467	662	888	512	606
6	12	452	143	231	202	< LOQ	219	181	< LOQ
7	12	405	234	193	< LOQ	< LOQ	< LOQ	< LOQ	< LOQ
8	14	1739	2465	1451	1442	1599	1595	1813	1248
9	20	452	129	183	110	< LOQ	< LOQ	< LOQ	< LOQ
10	18	405	236	147	< LOQ	< LOQ	< LOQ	< LOQ	< LOQ
11	18	178	102	< LOQ	< LOQ	< LOQ	< LOQ	< LOQ	< LOQ
12	20	178	< LOQ	< LOQ	< LOQ	< LOQ	< LOQ	< LOQ	< LOQ
13	20	290	168	< LOQ	< LOQ	< LOQ	< LOQ	224	233
14	20	5573	4670	5896	2296	2244	1769	1513	2054
15	18	850	845	389	365	522	474	301	310

**Fig. 1:**
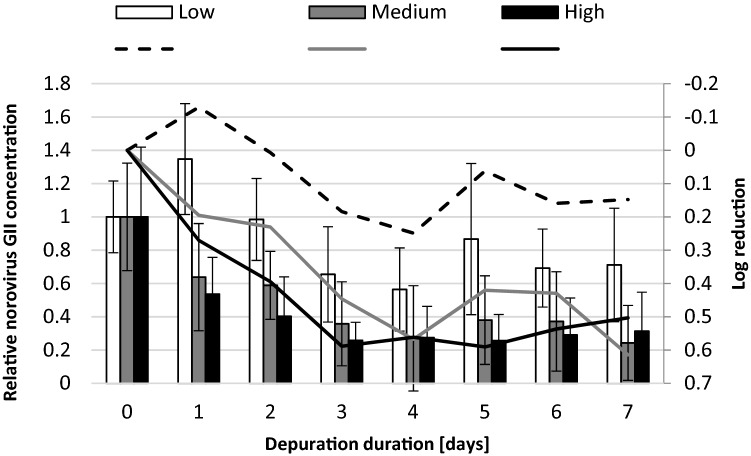
Log_10_ reduction (lines) of norovirus GII at 3 water temperature regimes studied and norovirus GII concentrations (bars) detected in oysters during laboratory depuration trials. Results shown as relative to norovirus GII concentration at day 0, error bars ± geometric standard deviation

Norovirus GII reduction in oysters during laboratory experiments are shown in Fig. 1. First indications of norovirus GII reduction in the medium and high temperature conditions were visible during the initial 24 h (Fig. 1). Virus reduction continued until day 3 at the high temperature conditions and until day 4 at the medium temperature conditions. Maximum reduction of norovirus GII was achieved by day 3 in the high temperature trials with relative virus concentration reduced to 0.26 ± 0.11 (reduction of 74% or 0.59 log_10_ units). A similar degree of reduction was observed in the medium temperature trials on day 4 with relative concentration reduced to 0.27 ± 0.32 (reduction of 73%, 0.57 log_10_ units). By contrast, norovirus GII concentrations were not reduced in oysters depurated under low temperature for the first 2 days of depuration. In fact, an increase in norovirus GII concentration was observed in these oysters at day 1 (relative concentration 1.35 ± 0.33) and the first signs of virus concentration reduction were only observed at day 3. The maximum reduction of the initial viral load to 0.65 ± 0.29 (reduction of 44% or 0.25 log_10_ units) was observed under low temperature conditions by day 4. Non-parametric Kruskal–Wallis ANOVA showed that there were significant differences in the norovirus GII concentrations observed between the low and high temperature (*p* < 0.001) and between low and medium temperature conditions (*p* < 0.001), but not between medium and high water temperatures (*p* = 0.14).

Norovirus GII reductions during all depuration trials conducted at medium and high water temperatures displayed a two-phase virus reduction kinetic. After the initial rapid reduction of norovirus GII, the rate of depuration decreased and no further significant reduction was observed between days 3 and 7 or days 4 and 7 for high and medium temperatures, respectively (Fig. 1). The two-phase virus reduction kinetic was not evident in the low temperature trials, with a delayed and shortened viral reduction stage.

No oyster mortalities were observed during the first 2 weeks of shelf life test conducted upon completion of the depuration experiments with all oysters responding to percussion. Mortality rates were comparable for the two oyster populations tested past week two, despite the different water temperature regimes used in depuration trials (paired *t* test *p* = 0.3724).

### Evaluation of Commercial Depuration

#### Producer A Commercial Depuration Results

A total of 13 paired samples (pre and post depuration) contaminated with norovirus GI, and 20 paired samples contaminated with norovirus GII, prior to depuration were identified from Producer A and included in this evaluation. The geometric mean concentrations in the pre-depuration samples were 251 gcg^−1^ and 281 gcg^−1^ and maximum concentrations detected were 761 and 1658 gcg^−1^ for norovirus GI and GII, respectively (Table 2). Following depuration, the geometric mean virus concentrations were < LOQ for both genogroups with the maximum concentrations of norovirus GI and GII detected in any sample 468 and 300 gcg^−1^, respectively. The difference in norovirus concentrations in oysters before and after depuration were found to be statistically significant for both genogroups (Mann–Whitney non-parametric *t*-test, *p* < 0.01 and *p* < 0.001, for norovirus GI and GII, respectively). A higher level of reduction was observed on average for norovirus GII compared to GI (Table 2) where norovirus GII was reduced by 83.95% (0.79 log_10_ units) compared to 67.58% (0.49 log_10_ units) reduction of norovirus GI.

**Table 2:** Norovirus GI and GII concentrations detected in oysters pre- and post-depuration collected from Producer A and B

Producer	A		B		
Depuration conditions	12–16 °C, 7 days		18 °C, 3 days		
Norovirus genogroup	GI	GII	GI	GII	GII^a^
*n* =	13	20	20	13	12
Pre-depuration [gcg^−1^]					
Average	285	344	242	844	806
Geometric mean	251	281	229	526	488
Min–Max	150–761	155–1658	151–409	167–2802	167–2802
Post-depuration [gcg^−1^]					
Average	121	65	129	317	158
Geometric mean	82	45	91	66	49
Min–Max	< LOD-468	< LOD-300	< LOD-366	< LOD-2232	< LOD-780^a^
Geometric mean reduction	67.58%	83.95%	60.41%	87.47%	89.92%
Log_10_ reduction	0.49	0.79	0.40	0.90	1.00

< LOD—results below the limit of detection (< 20 gcg^−1^ norovirus GI, < 20 gcg^−1^ norovirus GII)

^a^Analysis results on an adjusted sample population, with one outlier pair, failing to reduce norovirus GII concentration in oysters during depuration, excluded from norovirus GII results

#### Producer B Commercial Depuration Results

A total of 20 paired samples (pre- and post- depuration) contaminated with norovirus GI and 13 paired samples contaminated with norovirus GII were identified from Producer B and included in this evaluation. The geometric mean concentration in the pre-depuration samples were 229 gcg^−1^ and 526 gcg^−1^, for norovirus GI and GII, respectively (Table 2) with the maximum concentrations of 409 gcg^−1^ norovirus GI and 2802 gcg^−1^ norovirus GII. Following depuration, the geometric mean virus concentrations were significantly reduced to < LOQ for both norovirus GI and GII with maximum concentrations detected at 366 gcg^−1^ norovirus GI and 2232 gcg^−1^ norovirus GII. The difference in norovirus concentrations in oysters before and after depuration were found to be statistically significant for both genogroups (Mann–Whitney non-parametric *t*-test, *p* < 0.001 and *p* < 0.05 for norovirus GI and GII, respectively). In one of the 13 depuration runs analysed post depuration the concentration for norovirus GII (2232 gcg^−1^) did not decrease from the pre-depuration concentration of 1304 norovirus GII gcg^−1^. On removal of this outlier pair the overall level of reduction demonstrated for norovirus GII was almost 90% or 1 log_10_ units (*p* < 0.01). As observed for Producer A, a higher level of reduction was achieved for oysters contaminated with norovirus GII (87.47–89.92% or 0.90–1.00 log_10_ reduction) compared to norovirus GI (60.41% or 0.40 log_10_ reduction), despite the different depuration conditions employed.

Overall in the commercial settings, there was a significant difference in the level of reduction between norovirus GI and GII (*p* < 0.001) where norovirus GI was reduced on average by 63.4% (0.43 log_10_ units) compared to an on average reduction of 85.4% (0.84 log_10_ units) for norovirus GII (Table 3). The extent of reduction to < LOQ also differed between genogroups when the pre-depuration concentrations were < 1000 gcg^−1^ (*p* < 0.05). In this instance 51.5% of samples contaminated with < 1000 norovirus GI gcg^−1^ were reduced to < LOQ post-depuration, whilst 78.6% of samples contaminated with < 1000 norovirus GII gcg^−1^ were reduced to < LOQ (Table 3). Samples contaminated with norovirus GII at concentrations > 1000 gcg^−1^ prior to depuration were reduced to concentrations < LOQ in 40% of the cases. None of the samples examined in the commercial setting had concentrations of norovirus GI greater than 1000 gcg^−1^.

**Table 3:** Overall efficacy of commercial norovirus GI and GII depuration in Pacific oysters based on all paired results from Producer A and B, combined

Norovirus genogroup	GI	GII
Geometric mean reduction	63.40%	85.40%
Log_10_ reduction	0.43	0.84

Pre-depuration concentration	% Samples depurated to < LOQ	
< 1000 gcg^−1^	51.5	78.6
> 1000 gcg^−1^	n/a	40.0

## Discussion

Norovirus contamination in oyster production areas is an ongoing issue for food safety regulators and oyster producers alike. The primary objective for both is the production of food that is safe for human consumption. Data published in a recent EFSA baseline survey highlights the high prevalence of norovirus contamination in oysters from classified production areas during the winter months (EFSA 2019). Peak norovirus concentration and prevalence was observed in the months of January and February when almost 65% of samples tested were positive for norovirus with a mean concentration of 661 gcg^−1^ (EFSA 2019). This important data demonstrates the need for effective mitigation measures to reduce norovirus concentrations before reaching the consumer. Whilst there are currently no legislative criteria for norovirus in oysters intended for consumption, there is recognition from international food safety regulators for the need to introduce such a criterion to protect consumers. Therefore, oyster producers need effective virus reduction measures in order to meet any such microbiological criterion for norovirus in the future. Previously, we demonstrated the potential of risk management procedures to reduce the risk of norovirus contamination in a production area as well as the reduction of norovirus concentrations in end-product by depuration (Rupnik et al. 2018). In that particular study extended depuration periods of up to 9 days were applied during the high-risk winter season. In addition, during that study minimum depuration temperatures were generally above the recommended minimum temperature of 8 °C (Anonymous 2018) with the mean temperature over all depuration cycles of 13.3 °C during the winter. This prompted further investigation of the ability of enhanced depuration conditions to reduce norovirus concentrations in environmentally contaminated oysters destined for consumption. To our knowledge this is the first report of the application of a standardised test method (Anonymous 2017) to evaluate norovirus reduction during both, laboratory and commercial depuration processes to environmentally contaminated oysters.

The results of the laboratory depuration trials indicate that norovirus, whilst not completely removed, was significantly reduced when the water temperature used for depuration was increased to > 11 °C, but not at lower temperatures. Other studies have previously reported similar findings where norovirus was reduced, but not eliminated from contaminated shellfish, including clams, mussels and oysters (Ueki et al. 2007; Neish 2013; Polo et al. 2014a; Pilotto et al. 2019) using a variety of time and temperature combinations. The initial temporary increase in norovirus concentrations observed here at Day 1, and by others at Day 1–2 (McLeod et al. 2009; Polo et al. 2014c), could be explained by transient capture and subsequent release of norovirus particles from animal tissue outside of DT, such as gills and mantle (Maalouf et al. 2010). In addition, shellfish are known to slow down their metabolism and respiration rate at lower water temperatures (Dittman 1997; Li et al. 2017) and in such conditions relocation of virus particles from other tissues to DT and out as faeces or pseudofeces is less efficient and requires longer time.

The use of environmentally contaminated oysters in the depuration trials presented here, allowing for best representation of virus concentrations found in commercially harvested oysters, relied on long-term presence of the virus in environment in order to complete all planned experiments. Premature drop of norovirus GII concentration to levels below the critical lower limit (of 300 gcg^−1^) experienced during this study resulted in lower number of depuration trials completed at medium water temperatures, compared to low or high water temperatures. While such an adequately balanced data set could have provided improved, more robust statistics, the data set obtained during this study provides statistically significant differences between the different temperature regimes studied. Additional depuration studies, focusing on the medium water temperatures of 11 to 15 °C and using environmentally contaminated oysters, would be welcomed in the future to supplement the data presented here.

Initial viral load has been thought to have an impact on the depuration outcome whereby the higher the virus concentration prior to depuration, the higher the remaining virus load, if all other parameters were kept consistent (McLeod et al. 2017). The same finding was also observed in the study described here where oysters contaminated with < 1000 norovirus GII gcg^−1^, were reduced to < 100 gcg^−1^ in 1–4 days. However, the same post-depuration concentrations were not achieved in oysters contaminated with higher virus concentrations (> 1000 gcg^−1^). This study has demonstrated the impact of the initial viral load prior to commencing depuration trials on the variability in concentrations observed especially in the early stages (day 1–3). Nevertheless, the concentrations detected after day 3 in the medium and high temperature trials in many of the samples were at or below the LOQ for norovirus GII. This emphasises the importance of having knowledge of the starting concentration of norovirus in oysters prior to depuration and should be a key consideration for operators who wish to reduce norovirus concentrations to levels that may be below any future microbiological criterion.

A two-phase reduction of norovirus in oysters was observed during the laboratory depuration trials, similar to that reported in clams and mussels (Polo et al. 2014c). In this study once the maximum reduction was achieved after day 3 and day 4 no further significant decline in virus concentrations took place. A slight fluctuation of norovirus concentrations were detected during the second reduction phase (i.e. post day 3/4) in some experiments particularly on day 5 and 6. It is not clear what the exact cause is for these increases in virus concentration however it could be a reflection of the limitations of the RT-PCR method itself, especially when working with target concentrations close to, or below, the LOQ, a natural variability between samples or a potential re-contamination event due to sampling procedures. The optimal combination of time and temperature providing the most rapid reduction in norovirus was three days at the high temperature range with 74% (0.59 log_10_ units) reduction observed. However, a similar final reduction occurred in the medium water temperature range albeit one day later (73%, 0.57 log_10_ units). More limited norovirus reduction was achieved during depuration at the low temperature with maximum reduction of 44% (0.25 log_10_ units) observed after day 4. The rate of reduction observed in the increased temperature trials (medium and high) were significantly different from that observed from the low temperature trials. This indicates that by setting depuration water temperature to > 11 °C and by increasing the time allowed for depuration to 3–4 days, but not beyond, provides optimal reduction of norovirus from contaminated oysters whilst maintaining product shelf life.

To investigate norovirus reduction during depuration in the commercial setting we evaluated commercial practices undertaken by two separate oyster producers. Producer A employs longer depuration times (7 days) but lower water temperature (approximately 14 °C), while Producer B minimises the depuration time (3 days) but maximises the temperature (18 °C). In both scenarios, similar levels of viral reduction for norovirus GI and GII were achieved despite the difference in time and temperature combinations (Table 3). As with the laboratory trials the concentration of norovirus in the pre-depuration samples was an important feature of the overall success of commercial norovirus reduction as judged by a target concentration of < LOQ post-depuration. Almost four in five of all commercial oyster samples tested prior to depuration with a concentration < 1000 gcg^−1^ norovirus GII contained concentrations < LOQ post-depuration (Table 3). On the other hand, only 40% of samples with > 1000 gcg^−1^ prior to depuration, were found to have concentrations < LOQ for norovirus GII post-depuration. Despite a small number of commercial samples (*n* = 5) containing in excess of the 1000 gcg^−1^ norovirus GII included in this evaluation, combined with findings from the laboratory designed trials, these results suggest that whilst enhanced depuration conditions can reduce norovirus concentrations in oysters, this only becomes an effective consumer health intervention strategy when the contamination in the oyster harvesting area is also managed. Applying enhanced depuration conditions to oysters that are contaminated to levels > 1000 gcg^−1^, is unlikely to reduce norovirus concentrations to an acceptable level and will have limited public health benefit.

All but one laboratory depuration trial conducted at either medium or high water temperatures where the initial viral load in oysters was below 1000 gcg^−1^ yielded concentrations < LOQ after 4 days of depuration. In addition, one commercial depuration run (Producer B) also failed to reduce norovirus GII where the pre- and post-depuration concentration were similar. It is not clear what caused the poor depuration, or lack thereof, in these two instances but it does indicate that not all depuration runs will yield sufficient or similar reductions, despite consistent time and temperature conditions. This underlines the importance of monitoring the operation of commercial depuration systems to ensure reliable reduction of norovirus.

In both commercial depuration scenarios studied here the overall reduction rates were higher for norovirus GII than GI. Different behaviour between the viral strains during bioaccumulation and subsequent depuration have been reported previously (Nappier et al. 2008; Maalouf et al. 2011; Polo et al. 2014c) and could explain the differences observed here. Norovirus GI has been shown to bind to histo-blood like ligands in oyster digestive tissue, exhibiting prolonged persistence in oysters over GII norovirus indicating that its removal from oyster tissue may prove a greater challenge (Le Guyader et al. 2012). Laboratory trials as conducted for norovirus GII in this study are required for GI to fully elucidate the depuration kinetics for this genogroup. Unfortunately for the laboratory trials in this study, the production area from which the oysters were obtained does not have a history of regular norovirus GI contamination at concentrations suitable for such studies. Nonetheless, the results obtained from the commercial settings indicate the potential of enhanced depuration conditions to reduce norovirus GI concentrations to below the LOQ.

A greater reduction of norovirus GII was demonstrated in the commercial setting than in the laboratory trials with 84–87% (0.80–0.94 log_10_) reduction achieved by both commercial depuration processes compared with 74% (0.59 log_10_) in laboratory trials. The improved reduction observed in the commercial setting could be caused by a number of factors particularly those that affect the condition of the oysters themselves. Depuration is a complex process despite the simplicity of the infrastructure required. Individual animals can respond differently to stress factors, such as sudden changes in temperature, salinity, lack of food and physical disturbance (shaking, transport or rough handling) (Lacoste et al. 2001; Marigómez et al. 2017; Peteiro et al. 2018; Seuront et al. 2019). Distressed animals may have trouble resuming normal filtration and therefore may fail to purge impurities. Indeed, in all the laboratory trials visual inspection confirmed that oysters resumed filtration within an hour from submerging in water, suggesting minimal disruption. However, it is possible that a few animals were under some degree of distress during harvest, transport, upon submerging into heated water or while collecting the daily samples. In contrast to the laboratory setting, where a portion of oysters were removed from each depuration tank daily potentially unsettling the oyster population, the commercial depuration tanks were left undisturbed for the full duration of depuration (either 3 or 7 days). The tanks were drained once the process was completed and only then the oysters were removed, and the final post-depuration sample taken for analysis. This suggests the need for minimal handling and disturbance of the oysters during the depuration process to ensure greatest reduction.

Many studies investigating norovirus removal or reduction from bivalves have involved contaminating various shellfish species through bioaccumulation. Such laboratory contamination can result in very high starting concentrations which may not be reflective of concentrations naturally found in bivalves, especially oysters. Here, we used environmentally contaminated oysters from approved production areas to study norovirus reduction. Therefore, this represented concentrations of norovirus found in oysters prior to post-harvest treatment and destined for human consumption. The concentration prior to depuration in both the laboratory and commercial settings was significantly higher than the concentrations measured after depuration once the water temperature was > 11 °C. Of course, the question remains surrounding the public health significance of these remaining norovirus concentrations due to the lack of a routine norovirus infectivity assay despite promising developments with the human enteroid system (Ettayebi et al. 2016; Costantini 2018). The illness outcome for any given norovirus concentration in contaminated oysters will depend on the norovirus genotype, which could be single or multiple, the immune and genetic susceptibility of a host (Noda et al. 2008), as well as the size and number of oysters ingested by the consumer. However, a link between increasing number of virus genome copies detected and risk of infection has been reported previously (Lowther et al. 2012). A low likelihood of outbreaks was shown to be associated with oysters containing norovirus in concentrations below 100 gcg^−1^, levels readily achievable in this study in the depuration trials using increased water temperature and times. Hunt et al. (2020) demonstrated that norovirus GII was found to be log-normally distributed across individual animals within a population and that for mean concentrations below 100 gcg^−1^ it was likely that some of the oysters in the tested population contain no or very little amount of virus (< LOD). In contrast, all oysters in the tested population were found to be contaminated when the mean concentration tested above 300 gcg^−1^ (Hunt 2019). Additionally, two recent studies investigated the FRNA bacteriophage type II (norovirus surrogate) removal from oysters during depuration using genomic and infectivity methods (Leduc et al. 2020; Younger et al. 2020). These studies indicated that viruses may either be inactivated in oysters during depuration (Leduc et al. 2020) or, as suggested by Younger et al. (2020), destroyed and removed. Despite the differences in the concluded viral reduction mechanisms these findings could indicate that the virus genome copies detected using RT-PCR methods are, at least in part, only footprints left by the infectious virus particles and they no longer have the capability of causing infection especially at low concentrations (i.e. ≤ LOQ).

A recently published EFSA baseline survey provides comprehensive scientific data for norovirus prevalence in European oyster harvesting areas and will form the basis for discussion amongst EU authorities attempting to place a safety limit on norovirus concentration in oysters. Should a regulatory microbiological criterion for norovirus arise, reduction strategies such as enhanced depuration as described in this study may become an important intervention measure for food business operators to successfully comply with such a criterion. The application of enhanced depuration conditions, taking into consideration time and temperature, to environmentally contaminated oysters provides a practical tool for oyster producers enabling the reduction of norovirus concentrations to levels that reduce, if not eliminate, the risk to consumers. This study applied a standardised test method (Anonymous 2017) to verify norovirus reduction in both laboratory and commercial depuration trials providing reliable and robust data. Additionally, data arising from these experiments could be applied to mathematical prediction models, such as that developed by McMenemy et al. (2018) to predict the minimum time required to depurate norovirus to desired concentrations. Effectiveness of such forecasting models depends on comprehensive and reliable data. The combined effort of enhanced depuration for norovirus removal and reliable monitoring using a standardised method for detection of norovirus in shellfish may provide an important risk management tool for the oyster industry in light of potential forthcoming legislation.
